# DDR1 Regulates Femoral Arterial Calcification in Lower‐Extremity Artery Disease Through NF‐Kappa B Activation

**DOI:** 10.1111/apha.70146

**Published:** 2025-12-16

**Authors:** Manovriti Thakur, Thibaut Quillard, Nico Angliker, Mark Siegrist, Yvonne Jansen, Yi Yan, Julia Wollenhaupt, Claudia Goettsch, Lars Maegdefessel, Nadia Sachs, Marc Schindewolf, Drosos Kotelis, Heidi Noels, Yvonne Döring

**Affiliations:** ^1^ Division of Angiology, Swiss Cardiovascular Center, Inselspital, Bern University Hospital University of Bern Bern Switzerland; ^2^ Department for BioMedical Research (DBMR) University of Bern Bern Switzerland; ^3^ Nantes Université, CHU Nantes, CNRS, INSERM, L'institut du Thorax Nantes France; ^4^ Institute for Cardiovascular Prevention (IPEK) Ludwig‐Maximilians‐University Munich (LMU) Munich Germany; ^5^ Heart Center and Shanghai Institute of Pediatric Congenital Heart Disease, Shanghai Children's Medical Center, National Children's Medical Center Shanghai Jiao Tong University School of Medicine Shanghai China; ^6^ Institute for Molecular Cardiovascular Research (IMCAR) Uniklinik Aachen, RWTH Aachen University Aachen Germany; ^7^ Department of Biochemistry, Cardiovascular Research Institute Maastricht (CARIM) Maastricht University Maastricht the Netherlands; ^8^ Department of Internal Medicine I University Hospital of the RWTH Aachen Aachen Germany; ^9^ Institute of Physiology, Faculty of Medicine Carl Gustav Carus Technical University Dresden Dresden Germany; ^10^ Department for Vascular and Endovascular Surgery, TUM Klinikum Technical University Munich Munich Germany; ^11^ DZHK (German Centre for Cardiovascular Research) Partner Site Munich Heart Alliance Munich Germany; ^12^ Department of Vascular Surgery University Hospital Bern Bern Switzerland

**Keywords:** DDR1, femoral vascular smooth muscle cell, LEAD‐specific calcification, medial arterial calcification

## Abstract

**Aim:**

Lower‐extremity arterial disease (LEAD) is a manifestation of atherosclerotic cardiovascular disease, affecting 230 million people worldwide with increasing prevalence. Medial arterial calcification (MAC) is common in LEAD patients and contributes to disease‐related mortality. However, therapeutic strategies targeting femoral MAC are lacking, and its underlying mechanisms remain unclear. This study aimed to identify molecular drivers of femoral MAC in LEAD.

**Methods & Results:**

Calcium deposits and pro‐calcifying markers were analyzed in human patient samples using von Kossa staining, immunofluorescence, and gene expression analysis. Femorals showed significantly more calcification and pro‐calcifying gene expression than carotids. Given MAC abundance in LEAD, we assessed medial calcification in *Apoe−/−* mice fed a WD for 4/21 weeks. Digital PCR revealed upregulation of *Ddr1* and *Bmp2* in femoral versus carotid arteries after 21 weeks of WD. DDR1 expression positively correlated with calcification in human femoral samples. In vitro experiments with mouse femoral vs. carotid vascular smooth muscle cells (VSMCs) confirmed a significantly higher prevalence of calcifying proteins (DDR1, BMP2, and RUNX2) in femoral VSMCs. Additionally, calcification analyses in murine and human VSMCs showed that DDR1 inhibition reduced, while DDR1 activation increased, calcium deposition. Transcriptomic analysis revealed elevated NF‐κB expression in human femoral arteries, matching data in femoral VSMCs. DDR1 stimulation activated NF‐κB, and its inhibition blocked DDR1‐induced calcification.

**Conclusion:**

This study identifies DDR1 as a key driver of calcification in LEAD, operating through NF‐κB activation and the expression of calcifying proteins. Targeting DDR1 may offer a novel therapeutic approach to prevent MAC in LEAD.

## 1 Introduction

Lower‐extremity artery disease (LEAD), characterized by a narrowing and obstruction of arteries supplying the legs and feet, is a leading cause of cardiovascular diseases globally and is associated with significant morbidity and mortality. Although LEAD is one of the manifestations of atherosclerosis, it differs substantially from other atherosclerotic arterial diseases, especially with respect to calcification [1]. Calcification predominantly affects the intimal layer of atherosclerotic coronary arteries, common carotid arteries, and aorta [2], whereas arteries of the lower extremities in LEAD patients typically display calcification in the medial layer of the arteries—referred to as medial arterial calcification (MAC), also in non‐atherosclerotic arteries [3].

Distinct from atherosclerotic plaque formation, MAC, also known as Mönckeberg's arteriosclerosis, plays an independent role in LEAD progression [4]. Overall, a study of 176 arteries from amputated legs of patients with critical limb ischemia found MAC in 72% of femoral arteries [3]. Similarly, another study showed that most occluded vessels in critical limb ischemia amputations exhibited MAC rather than plaque buildup [5].

The severity of MAC in LEAD, assessed through the Pedal MAC score, is associated with an increased risk of lower limb amputation in chronic limb‐threatening ischemia patients [6]. Furthermore, MAC in peripheral arteries is independently associated with increased arterial stiffness [7, 8], with MAC inducing significant structural changes within the vessel wall, thereby reducing vessel compliance and altering blood flow dynamics [9]. MAC‐induced arterial stiffness has also been associated with worse outcomes following lower‐extremity interventions for limb‐threatening ischemia [9]. Arterial stiffness can complicate endovascular repair using balloon angioplasty, as high‐pressure dilatation can cause medial dissection and neointima formation. Despite its clinical significance, the molecular mechanisms underlying LEAD‐specific MAC and arterial stiffness remain poorly understood [3, 4].

Studies on molecular mechanisms driving MAC suggest that BMP2, a bone morphogenetic protein, and Runx2, a master regulator of osteoblast differentiation, influence vascular smooth muscle cells (VSMCs) to undergo a phenotypic switch toward osteogenic/chondrogenic cells, ultimately leading to calcification [10, 11]. In medial calcification, the process of differentiation of smooth muscle cells (SMCs) into osteoblast‐like cells is akin to bone formation and is related to genes such as *BMP2*, *Msh Homeobox 2* (*MSX2*), and alkaline phosphatase (*ALP*) [11]. Interestingly, most calcified arteries have higher levels of BMP2 and increased BMP2‐induced VSMC proliferation compared with noncalcified arteries [10]. Elevated levels of BMP2 promote phosphate uptake and calcification of human VSMCs [10]. Knockdown of *Runx2* in VSMC inhibits osteogenic conversion and matrix mineralization [12].

The collagen‐binding receptor tyrosine kinase ‘Discoidin Domain Receptor‐1’ (DDR1) has emerged as a key regulator of vascular calcification. DDR1 deficiency is shown to reduce aortic arch calcification through VSMCs‐mediated mineralization during atherosclerosis (PMID: 19893047 [13]). DDR1 has previously been shown to regulate RUNX2 in osteoblasts, and *Ddr1* knockout leads to decreased mineralization and decreased protein levels of BMP2, RUNX2, and ALP during osteogenesis [14]. Additionally, DDR1 global knockout has been shown to impact bone microarchitecture and mechanics in aging female mice, highlighting its role in bone and mineral homeostasis 39 776 614 [15]. Furthermore, tumor necrosis factor (TNF‐alpha)‐induced activation of NF‐κB (nuclear factor‐kappa B), a transcription factor involved in inflammation and immune responses, has also been shown to accelerate vascular calcification [16]. Whether these regulators and signaling pathways also contribute to femoral arterial calcification in lower extremities remains currently unknown. Therefore, we investigated the molecular players and pathways specific to LEAD‐associated calcification. This study identifies molecular mechanisms involved in femoral medial calcification during hyperlipidemia‐induced inflammation, a known driver of MAC [17] in *Apoe−/−* mice and LEAD patients, and offers novel potential therapeutic targets.

## 2 Material and Methods

All the materials submitted conform with good publishing practice in physiology according to Acta Physiologica guidelines.

### 2.1 Patient Samples

Carotid artery tissues (10 males and 10 females) and femoral arterial tissues (5 males and 5 females) were obtained from the Munich Vascular Biobank. Sample collection adhered to the Declaration of Helsinki and was approved by the local ethics committee of the Technical University of Munich (approval number: 2799/10). Informed written consent was obtained for all patients. Clinical data was retrieved from electronic patient records. Available clinical and demographic information for the human subjects from whom carotid and femoral artery samples were obtained summarizes age, sex distribution, hypertension, chronic heart disease, diabetes status, ASA classification of the patient's physical status, renal function (dialysis), BMI, and statin use (Table S1). Tissue samples were fixed in 4% paraformaldehyde (PFA) for 24 h and decalcified using Entkalker soft SOLVAGREEN (Carl ROTH, Karlsruhe, Germany) for 5 days. Decalcified specimens were then embedded in paraffin and stored as formalin‐fixed paraffin‐embedded (FFPE) blocks for further processing. Sections of paraffin‐embedded samples were mounted on glass slides (Menzel, 76 × 26 × 1 mm, Fisher Scientific, Schwerte, Germany).

Furthermore, 10 other samples (5 carotids and 5 femoral) were obtained through the ECLAGEN program at the Nantes University Hospital. The ECLAGEN biocollection includes human carotid bifurcation and common femoral artery samples. Details of this biocollection have been described in previous publications [18]. Pathological atheromatous tissues were collected during endarterectomy, and healthy arteries were collected from organ donors. In the operating room, the atheromatous lesions coming from the endarterectomy were cut into two pieces, one for RNA extraction and molecular analysis, and the other for histological analysis. Stratification of lesion calcification was performed after histomorphometry analysis of mineralized structures, as depicted in our previous studies [19]. The collection of human samples was carried out according to strict ethical standards. All living participants received an information notice and signed a written consent (research protocol #PFS09‐014, authorized on December 23, 2009, by the French “Agence de Biomédecine”). For deceased donors, no opposition to organ donation was checked, and written consent from the donor's family was obtained. The legal authorizations were obtained from the French Ministry of Research (nDC‐2008‐402), the National Commission for Informatics and Freedoms (CNIL, n1520735v0), and the Local Ethics Committee (GNEDS, Groupe Nantais d'Ethique dans le Domaine de la Santé).

### 2.2 Mice

All animal experiments were approved by the local ethical committee (Amt für Veterinärwesen, Kanton Bern) with national approval number: BE72/2023. *Apoe−/−* mice were bred in the local animal facility under SPF status and fed a normal diet (KLIBA NAFAG 3437) before the start of the experimental diet. For the initiation and development of atherosclerosis, mice were fed a western‐type diet (WD) containing 21% fat and 0.15%–0.2% cholesterol (Ssniff TD88137), starting at 8–10 weeks of age for 4 or 21 weeks.

### 2.3 Cell Culture

Murine carotid artery SMCs (Creative bioarray, Cat. No. CSC‐C5356S) and murine femoral artery SMCs (Creative Bioarray, Cat. No. CSC‐C5361S) were grown in SuperCult Mouse Smooth Muscle Cell MediumSuperCult (Creative bioarray, Cat. No. CM‐2008S) at 37°C in humidified 5% CO_2_ in T25 (Corning, Cat. No. CLS430639‐200EA) and T75 flask (Corning, Cat. No. CLS430641‐100EA). Upon reaching 80%–90% confluence, cells were trypsinized with trypsin/EDTA (Lonza, CC‐5034) and passaged. Cells were used between passages 3 and 6. Cells (100 000 per well) were seeded in a 6‐well plate, and after 80% confluency, they were incubated with normal media (NM) consisting of DMEM high glucose (Gibco, 11 965 092) + 1% PS (Gibco, Cat. No. 15140122) + 10% FBS (Sigma, Cat. No. F9665‐50ML), and calcifying media (CM) consisting of NM + 2.8 mM Phosphate (by adding Sodium dihydrogen phosphate (NaH_2_PO_4_) and disodium hydrogen phosphate (Na_2_HPO_4_) in a 1:1 ratio) [20] for 7 days in a humidified, 5% CO2 atmosphere. The culture media was changed every 48–72 h. For DDR1 stimulation, we used soluble 10 μg/mL collagen type I (Sigma Aldrich, Cat. No. C3867), and for inhibition, we used 1 μM per well DDR1‐IN‐1 (Sigma Aldrich, Cat. No. SML2274‐25MG) in culture medium with replacement every 48 h. NF‐κB‐p65 was inhibited using 1 ng/mL of MG132 (Sigma Aldrich, Cat. No. 474790‐1MG), which was added from day zero, and media was replaced every 48 h. After a total incubation period of 7 days, the VSMCs were washed twice with phosphate‐buffered saline (PBS), and the VSMCs were lysed with HCl (0.1 mM; pH 3; 120 μL per well) at 4°C for 60 min, followed by colorimetric calcium analyses.

### 2.4 Isolation of Femoral and Carotid VSMCs

Cells were isolated from murine femoral and carotid arteries, where small sheets of the media layer were stripped from the luminal surface of the arteries, cut into smaller segments (∼1 mm), and collected in DMEM media supplemented with 10% FBS and 1% penicillin/streptomycin. These pieces were then spaced evenly onto dry, sterile, uncoated culture plates and allowed to be attached by dry adhesion for 10 min before being covered by supplemented DMEM media and placed in an incubator. Media changes every 2 days resulted in the eventual outflow of migratory SMCs and progenitors by ∼10 days. The original arterial strips were then removed, and the remaining migratory cells were allowed to become confluent in the incubator. At confluency, these cells were transferred to larger flasks and returned to the incubator with the media changed every 2 days until confluency. These P2 cells were then counted, characterized, and stored at −150°C as aliquots containing 500 000 cells. For human VSMC isolation, briefly, human carotid and femoral VSMC were isolated from organ donors with no macroscopic atherosclerosis lesions, and most of the healthy arteries were from accidental cases. The exclusion criteria were as follows: Adults under guardianship or conservatorship, pregnant women, minors, restenosis, non‐atheromatous lesions (dysplasia, posttraumatic, inflammatory), thrombosis, history of cardiovascular disease (ischemic heart disease, stroke, peripheral artery disease), arteries showing macroscopic signs of atherothrombosis during sampling, and participation in another therapeutic trial. Smooth Muscle Cell Growth Medium 2 (C‐22062) from Promocell was used to grow the cells. To run the mineralization experiments, the cells were cultured in DMEM with 3% FBS in NM or CM.

### 2.5 Cell Viability Assay

AlamarBlue Cell Viability Reagent (Invitrogen, Cat. No. DAL‐1025) was used to check the viability of the VSMCs in the 7‐day cell culture experiments with NM or CM and with DDR1 stimulator (collagen type‐1, 10 μg/mL), DDR1 inhibitor (DDR1‐IN‐1, 1 μM), and NF‐κB‐p65 (MG132, 1 ng/mL). 50 μL of 10X Alamar blue reagent was added to the 450 μL of appropriate cell culture media, followed by incubation for 1 to 4 h at 37°C in a cell culture incubator, protected from direct light. Thereafter, the absorbance of AlamarBlue was measured at 570 nm, using 600 nm as a reference wavelength (normalized to the 600 nm value) using Varioskan Lux plate reader (Thermo Scientific) and Skanlt RE 7.0.2 software.

### 2.6 Histology and Immunofluorescence

Human carotid artery and femoral artery tissues (20 and 10 samples) were embedded in paraffin. Sections (5 μm) were deparaffinized, rehydrated, and then subjected to heat antigen retrieval in 0.01 mM sodium citrate buffer (pH 6.0) and inactivation of endogenous peroxidase with 3% H_2_O_2_, and used further for von Kossa or immunofluorescence staining. For calcium/mineralization detection, von Kossa staining was performed using Von Kossa calcium staining kit (Sigma Aldrich, Cat no: 1065590500). The tissue slides were incubated with silver nitrate solution (5%; 500 μL) in the presence of UV light (405 nm) for 1 h and washed three times with ddH_2_O (1 mL each), incubated in the presence of aqueous sodium thiosulfate (5%; 500 μL) for 1 min, and washed three times with ddH_2_O (1 mL each). Thereafter, tissue slides were incubated with nuclear fast red solution (Cat no. N3020, 500 μL) at room temperature for 5 min and rinsed with H_2_O (1 mL). Slides were covered with Vitro‐Clud solution (500 μL), and digital photographs of the stained arterial rings can be taken using a Nikon Eclipse Ti‐E microscope. Fiji 2.9.0 software was used to quantify the calcified area of the artery. The first region of interest was selected, and the same threshold was set for all the samples from the same day of staining.

For immunofluorescence staining, after fixation, blocking was performed with 5% BSA, sections were then incubated with primary antibodies RUNX2 (1:1900 dilution, NovusBio, NBP1‐77461), BMP2 (1:50 dilution, Invitrogen, PA5‐85956), DDR1 (1:50, Invitrogen, PA5‐64780), annexin V (BD, 560931), NF‐κB p65 (1:100, Cell Signaling, 3033 T) overnight at 4°C. After incubation with a secondary FITC‐ or Cy3‐conjugated or Alexa fluor 488, 594, 647 antibody (Life Technologies) for 60 min at room temperature, and nuclei were counter‐stained with 4′,6‐Diamidino‐2‐phenylindole (DAPI), and the sections were embedded with Immu‐Mount (Thermo Scientific), and images were made using a confocal laser scanning microscope LSM 980 (Zeiss) and Zen 3.3 software. In our analysis, we ensured that comparable regions of the vascular wall, that is, the medial layer was examined across carotid and femoral artery specimens.

Similarly, for cell culture assays, 1000 cells per well were seeded in 12‐well ibidi chambers and incubated in NM or CM with or without DDR1 stimulator, DDR1 inhibitor, and NF‐κB inhibitor for 7 days. Thereafter, supernatant was removed, and cells were fixed in 1% Paraformaldehyde (PFA) for 10 min followed by 3 washes with PBS and blocking with 2% BSA for 60 min. Wells were then washed 3 times with PBS and incubated with primary antibodies RUNX2 (1:1900 dilution, NovusBio, NBP1‐77461), BMP2 (1:50 dilution, Invitrogen, PA5‐85956), DDR1 (1:50, Invitrogen, PA5‐64780), annexin V (BD, 560931), NF‐κB p65 (1:100, Cell Signaling, 3033 T) overnight at 4°C. After incubation with a secondary FITC‐ or Cy3‐conjugated or Alexa fluor 488, 594, 647 antibody (Life Technologies) for 60 min at room temperature, nuclei were counter‐stained with 4′,6‐Diamidino‐2‐phenylindole (DAPI), and the sections were embedded with Immu‐Mount (Thermo Scientific), and images were made using confocal laser scanning microscope LSM 980 (Zeiss) and Zen 3.3 software.

Analyses of the images were performed using Zeiss Zen 3.7, Imaris 9.6, and Fiji 2.9.0 software. For images from the von Kossa staining and immunofluorescence staining of human and mouse arterial tissues, first the area of interest was selected by outlining the medial layer of the arteries. RUNX2 and BMP‐2 staining were quantified using ImageJ. Nuclear RUNX2 area was determined by co‐localizing DAPI‐stained nuclei and RUNX2 signals. BMP‐2 signal area was quantified based on intensity thresholding using consistent parameters across images. For mouse samples, a minimum of five random regions of interest (ROIs) per sample were analyzed. Also, for human samples, analyses were done on five randomly selected images within one sample. Values of the different ROIs were combined and averaged to generate one value per sample. Data was represented as a calcifying area or a positively stained area per visual field.

### 2.7 Calcium Content and Protein Analyses

Calcium content was quantified by the Calcium calorimetric assay kit (Sigma Aldrich, Cat No. MAK477). VSMCs in a 12‐well plate were treated with NM or CM for 7 days and then decalcified with 0.1 M HCl for 1 h at 4°C. Alternatively, this assay was also performed with carotid and femoral arterial rings harvested from *Apoe−/−* mice. Briefly, carotid and femoral arteries were harvested from *Apoe−/−* mice, cleaned of connective tissue, and cut into rings. These rings were cultured in NM and CM for 7 days and then decalcified with 0.1 M HCl for 24 h at 4°C. The supernatants were centrifuged for the calcium content assay according to the kit manufacturer, and the absorbance was measured at a wavelength of 612 nm. In addition, the cells were solubilized in 0.1 M NaOH and 0.1% SDS, and their protein concentrations were determined using a Pierce BCA Protein Assay Kit (Thermofisher, Cat No. 23227) according to the kit manufacturer. Finally, the calcium contents were normalized to total protein concentrations.

### 2.8 ELISA

For NF‐κB p65 and Phospho‐ NF‐κB p65 ELISA, the cells were harvested in Cell Lysis Buffer (Cell Signaling cat. no. 9803) with 1 mM PMSF (Sigma Aldrich cat. no. P7626) added after having washed the cells with PBS. The cells were fully harvested using a cell scraper and subsequently sonicated on ice. After centrifugation at 18 000 rcf for 10 min at 4°C, the supernatant was transferred to a new tube, and the protein concentration was measured using a Pierce BCA Protein Assay Kit (ThermoFisher cat. no. 23227). ELISA kits from Cell Signaling were used for the measurement of NF‐κB (cat. no. 7174) and Phospho‐NF‐κB (cat. no. 7173) according to the manufacturer's instructions. For both ELISAs, cell lysate was diluted in the ELISA Sample Diluent of the kit to a concentration of 75 μg/mL. Absorbance was finally measured at 450 nm using a Varioskan Lux plate reader (Thermo Scientific) and Skanlt RE 7.0.2 software.

### 2.9 Western Blot

Protein was collected with 1× RIPA lysis buffer (cat. no. 20‐188, Merck) supplemented with protease inhibitor (cOmplete cat. no. 05892791001, Roche) and phosphatase inhibitor (PhosSTOP cat. no. 4906845001, Roche) from cells grown in 6‐well plates. Protein concentration was measured by Pierce BCA Protein Assay Kit (cat. no. 23227, Thermofisher). Equal amounts of protein were added to 4 × Laemmli Sample Buffer (cat. no. 1610747, Biorad) supplemented with 10% (v/v) β‐Mercaptoethanol, boiled for 5 min, and separated by SDS‐PAGE at 100 V. The protein was transferred onto a polyvinylidene fluoride (PVDF) membrane using the iBlot 2 Western Blot Transfer System of Thermofisher (cat. no. IB24001, ThermoFisher). Membranes were blocked for 1 h with Intercept (PBS) Blocking Buffer (cat. no. 927‐70001, LI‐COR). Primary antibodies were diluted in Intercept (PBS) Blocking Buffer + 0.2% Tween 20 (v/v) or Tris‐buffered saline + 0.1% Tween 20 (v/v) + 5% BSA (w/v) for phosphoproteins, respectively, and incubated with the membranes at 4°C overnight (Antibody dilutions see below). DyLight 800—labeled secondary antibody (cat. no. SA5‐10036, Thermofisher) diluted 1:10 000 in Intercept (PBS) Blocking Buffer/PBS (1:1) +0.2% Tween 20 (v/v) was applied for 1 h at RT in the dark, and for imaging and quantification of bands, the Li‐Cor Odyssey Infrared Imaging System was used. Antibodies used for Western blot were beta‐Actin (13E5) (cat. no. 4970S, Cell signaling, 1:1000), Phospho‐NF‐κBp65 (Ser536) (93H1) (cat. no. 3033 T, Cell signaling, 1:1000), NF‐κB p65 (cat. no. ab16502, Abcam, 1:2000).

### 2.10 RNA Isolation and Digital PCR

RNA isolation of murine femoral and carotid arteries was performed using the RNeasy Fibrous Tissue Mini Kit of Qiagen (cat. no. 74704) according to the manufacturer's protocol. Femoral arteries from *Apoe−/−* mice are smaller and yield less total RNA compared to carotid arteries. As a result, in certain cases, different n numbers (biological replicates) were used to perform gene expression analyses for the calcifying genes in *Apoe−/−* mice due to limited RNA/cDNA yield from some femoral artery samples, which restricted the number of genes we could reliably analyze per sample. Prior to RNA isolation, arteries had been stored in RNAlater (cat. no. R0901‐100ML, Merck), and tissue was lysed using stainless steel beads (cat. no. 69989, Qiagen) in combination with the Tissue Lyzer II of Qiagen. For RNA isolation of cultured cells, the AllPrep DNA/RNA/Protein Mini Kit (50) of Qiagen was used (cat. no. 80004) according to the manufacturer's protocol. Isolated RNA was quantified using a NanoDrop 2000 Spectrophotometer (Thermo Scientific), and equal amounts were subsequently reverse transcribed to cDNA using the iScript cDNA Synthesis Kit of Biorad (cat. no. 1708891). Droplet digital PCR (ddPCR) was performed on the Applied Biosystems Absolute Q Digital PCR System (Thermo Fisher Scientific) or on a Biorad QX200 ddPCR system according to the manufacturer's instructions using DNA Digital PCR Master Mix (5×) (cat. no. A52490, Thermofisher) or ddPCR Supermix for Probes (cat. no. 1863010, Biorad), respectively (see table below). Details on probes and amounts of input DNA are specified in the table below.

**Table:** 

Gene	Label	Assay ID	Manufacturer	ddPCR system used	Input cDNA	Source of cDNA
*Ddr1*	FAM	Mm01273496_m1	Thermofisher	Absolute Q	60 ng (for cells) 9 ng (for tissue)	*Cells*: Cultured murine smooth muscle cells (femoral or carotid) *Tissue*: Murine femoral and carotid artery
*Alpl*	VIC	Mm00475834_m1	Thermofisher	Absolute Q		
*Runx2*	ABY	Mm00501584_m1	Thermofisher	Absolute Q		
*Bmp‐2*	FAM	Mm01340178_m1	Thermofisher	Absolute Q		
*Rela*	FAM	Mm00501346_m1	Thermofisher	Absolute Q		
*Gapdh*	HEX	dMmuCPE5195283	Biorad	Absolute Q	0.6 ng (for cells) 0.09 ng (for tissue)	
*Ddr1*	HEX	dMmuCPE5093873	Biorad	Biorad QX200		
*Gapdh*	HEX	dMmuCPE5195283	Biorad	Biorad QX200		

### 2.11 Human Gene Expression Microarray Dataset Analysis

To examine the *BMP2, ALPL, RUNX2*, and *DDR1* mRNA expression in atherosclerotic and control tissues from peripheral arteries of carotid or femoral territories, one microarray dataset was obtained from Gene Expression Omnibus (www.ncbi.nlm.nih.gov/geo) under the accession number GSE100927. In this dataset, a total of 79 samples were included, composed of 12 control carotid arteries without atherosclerotic lesions, 12 control femoral arteries without atherosclerotic lesions, 26 atherosclerotic lesions from carotid arteries, and 29 atherosclerotic lesions from femoral arteries [18]. Data preprocessing included transforming gene probes into gene symbols, data consolidation, and normalization. Probes without gene symbols were deleted. Probes with maximal expression were retained for further analysis if the probes contained more than one probe. Next, differentially expressed genes (DEGs) between atherosclerotic femoral and control tissues were identified as previously described [21]. In brief, the limma R package was used to perform the differential gene analysis. The following selection criteria were used to screen out DEGs: fold change > 1.5 or < 0.67 between two groups and adjusted *p* value (calculated by Benjamini and Hochberg's method) < 0.05. Transcription factors regulating 147 upregulated genes in femoral atherosclerotic lesions compared to atherosclerotic lesions from carotid arteries were predicted based on the database called Transcriptional Regulatory Relationships Unraveled by Sentence‐based Text mining (TTRUST) and visualized in a bar plot.

### 2.12 Statistical Analysis

Data was expressed as mean ± SEM. Data was tested for a normal (Gaussian) distribution using the Shapiro–Wilk normality test and the Kolmogorov–Smirnov normality test with Dallal‐Wilkinson‐Lillie for the *p* value before applying the unpaired Student's *t*‐test between two groups for normally distributed data or the Mann–Whitney test for nonparametric data. Comparison of more than two groups was performed by ordinary one‐way analysis of variance (ANOVA) followed by Tukey's multiple comparisons post test, or by the Kruskal–Wallis test for nonparametric data. Analysis of more than two groups with multiple variables was performed using a two‐way multivariate ANOVA. For correlation analyses, Spearman's correlation test was performed. All statistical analyses and figures were performed using GraphPad Prism 9.0 Software. Each data point in the graph represents an independent donor or biological replicate. *p* < 0.05 was considered statistically significant.

## 3 Results

### 3.1 Femoral and Carotid Artery Calcification Differ During Chronic Arterial Inflammation

**FIGURE 1:**
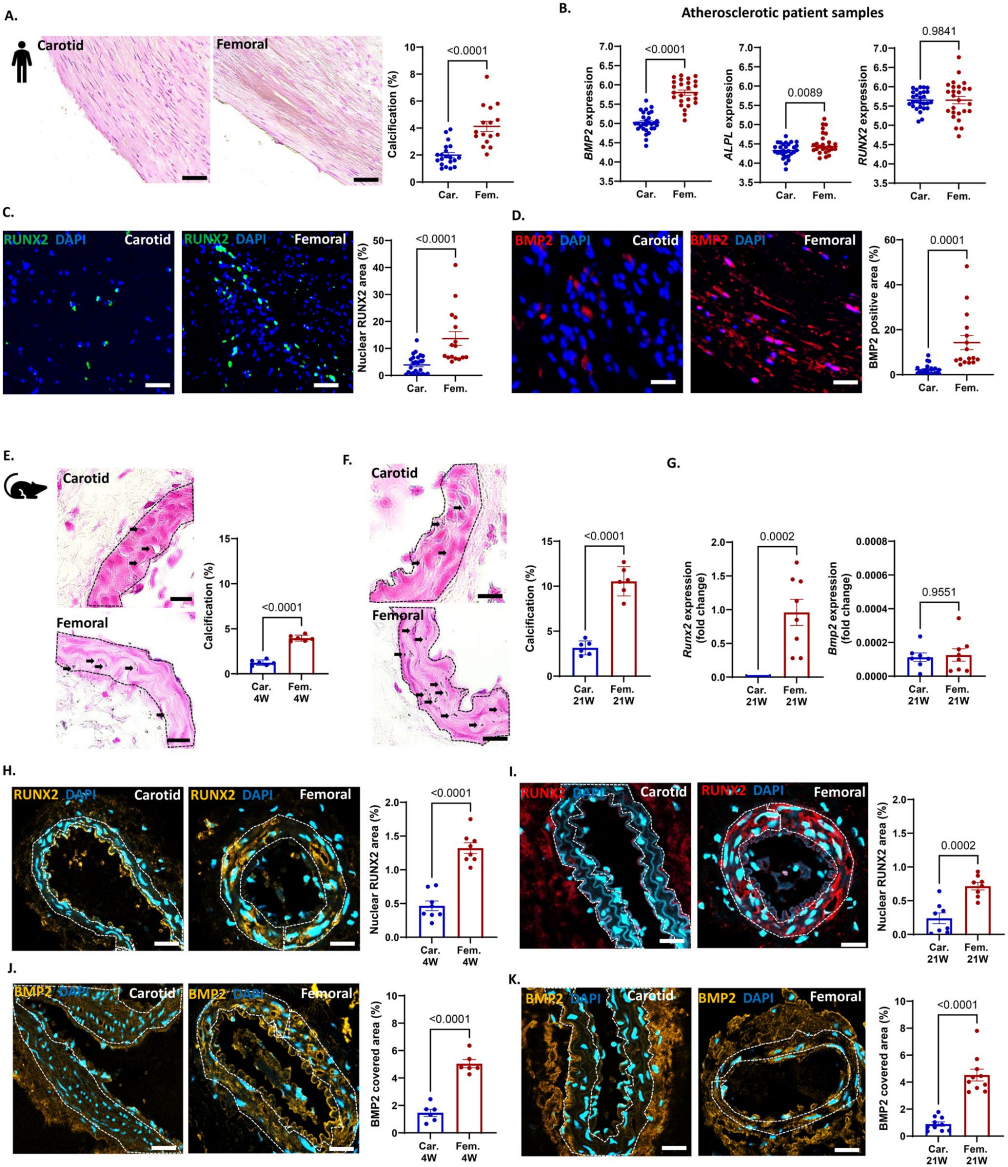
Femoral calcification differs from carotid calcification during arterial inflammation. (A) von Kossa staining and quantification of mineralization in carotid and femoral samples from atherosclerotic patients; analyses depict calcification percentage per visual field. Scale bar = 100 μm. (B) Quantification of calcifying gene expression (fold change) of *BMP2, ALPL* and *RUNX2* in atherosclerotic carotid (*n* = 27) and femoral artery (*n* = 25) samples from patients (dataset GSE100927). (C, D) Immunofluorescent imaging and quantification of (C) RUNX2 and (D) BMP2 in carotid and femoral endarterectomies from atherosclerotic patients. Scale bar = 50 μm. (E, F) von Kossa staining and quantification of mineralization in the medial layer of carotid and femoral arteries of *Apoe−/−* mice fed with (E) 4 weeks and (F) 21 weeks of WD. Scale bar = 20 μm, *n* = 6 per group. (G) Expression of *Runx2* and *Bmp2* (normalized to *Gapdh*) in carotid and femoral arteries of *Apoe−/−* mice on 21 w of WD using digital droplet PCR, *n* = 8 per group. (H–K) Immunofluorescent imaging and quantification of RUNX2 (nuclear associated) and BMP2 covered area in the medial layer (excluding endothelial and adventitial areas) of carotids and femorals of *Apoe−/−* mice with 4 weeks or 21 weeks of WD. Scale bars = 20 μm, *n* = 8 per group. Dot plots (for human samples) and Bar graphs (for murine samples) represent mean ± SEM. Each dot/point on the graph represents one animal or one patient sample. Unpaired student *t*‐test and Mann–Whitney test are used to compare the groups. *p* values as depicted, values ≤ 0.05 are considered significant.

To investigate calcification differences during atherosclerosis in different vascular beds, we performed mineralization analyses using von Kossa staining on carotid vs. femoral samples obtained from patient thromboendarterectomy specimens. The clinical and demographic information for the human subjects from whom carotid and femoral artery samples were obtained is summarized in Table S1. Our results from von Kossa staining revealed significantly higher calcification in atherosclerotic femoral samples compared to carotid samples (Figure 1A). We further examined the mRNA expression of key calcifying proteins in human and mouse samples. Human gene expression data (GSE100927) [18] from carotid (*n* = 27) and femoral (*n* = 25) specimens showed significantly higher *BMP2* and *ALPL* expression in femoral arteries (Figure 1B). No significant changes were observed in arterial samples obtained from non‐atherosclerotic human donors (Figure S1A). Even though no changes were observed in *RUNX2* gene expression, a higher prevalence of RUNX2 protein was observed in femoral compared to carotid arteries using immunofluorescent staining (Figure 1C). Also, a significant increase in BMP2 protein levels was observed in the femoral thromboendarterectomy specimens (Figure 1D).

It is described that *Ldlr−/−* and *Apoe−/−* mice do not develop femoral intimal atherosclerotic lesions after 21 weeks of WD feeding [18]; however, as our focus was on MAC, which plays a central role in lLEAD (see graphical abstract), we still considered hyperlipidemic *Apoe−/−* an appropriate model to investigate MAC [17]. Consequently, in our histological analyses, we focused on medial areas of the vessel wall, excluding intimal plaque cores where possible, to capture MAC and avoid confusion by intimal (atherosclerotic) calcification. To this end, we placed *Apoe−/−* mice on WD and analyzed calcification in the medial layer of femoral and carotid arteries of mice fed a WD for 4 and 21 weeks. Our results demonstrated significantly increased medial layer mineralization in the femoral compared to carotid arteries (Figure 1E,F). Correspondingly, digital droplet PCR analyses of murine femoral arteries revealed significantly increased *Runx2* expression in the femoral arteries compared to carotid arteries of *Apoe−/−* mice fed a WD for 21 weeks (Figure 1G). Although there were no significant differences in gene expressions of *Bmp2* in *Apoe−/−* mice (21w WD) and *Runx2*, *Bmp2* and *Alpl* in femoral vs. carotid arteries of *Apoe−/−* mice (4w WD) (Figure S1B), increased nuclear associated RUNX2 and BMP2 protein prevalence was detected in the medial layer of femoral arteries of *Apoe−/−* mice with 4w WD (Figure 1H,J) and 21w WD (Figure 1I,K). Additionally, tissue nonspecific alkaline phosphatase (TNAP) was also more prevalent in the medial layer of femoral arteries compared to carotid arteries (Figure S1C,D). Together, these data confirm vascular bed‐specific differences regarding calcification potential, with femoral arteries and femoral VSMCs being more prone to calcification as compared to the carotid samples.

### 3.2 Increased Calcification Potential of Femoral Compared to Carotid VSMCs

**FIGURE 2:**
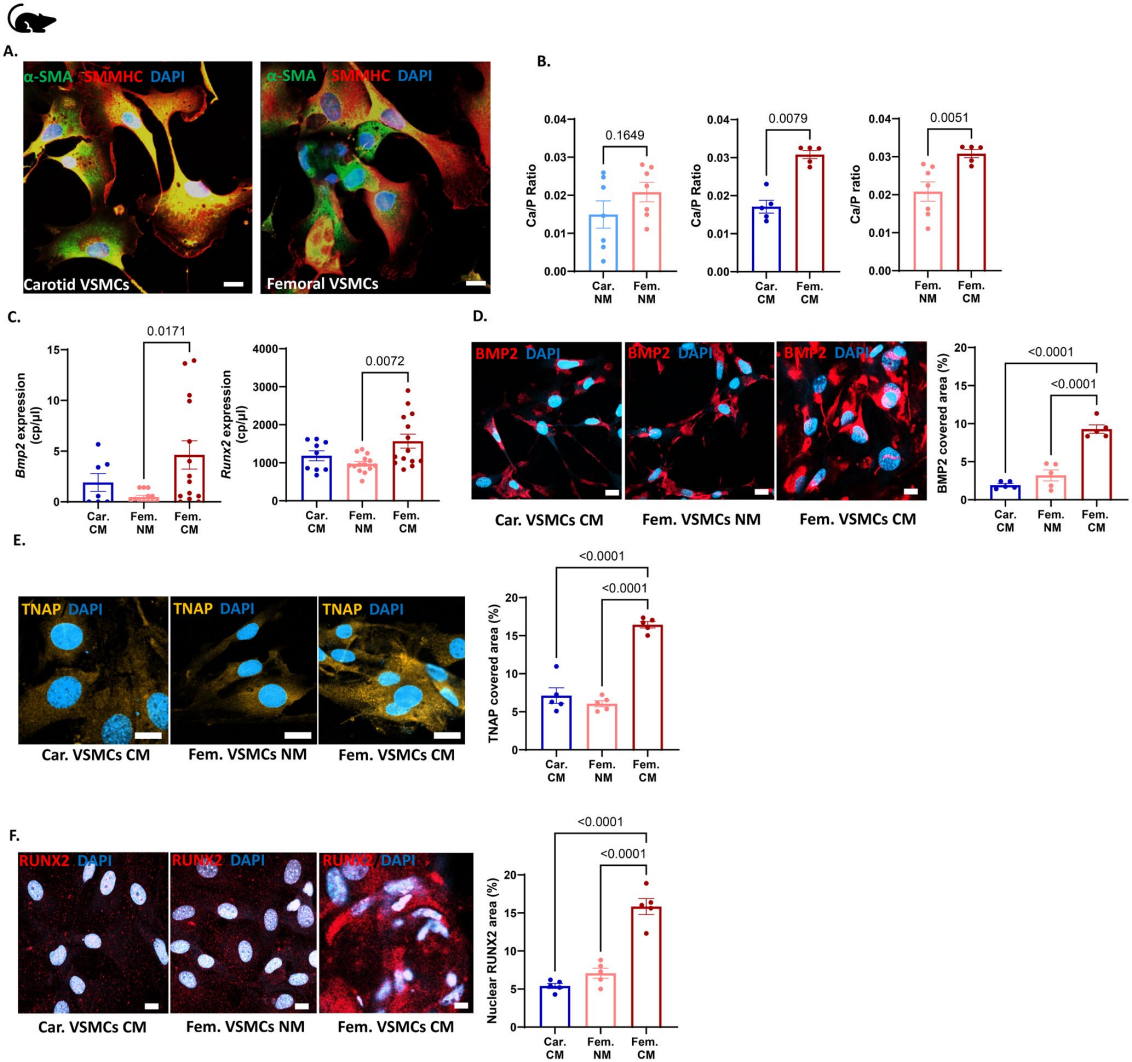
Femoral calcification differs from carotid calcification in vitro. (A) Characterization of cultured VSMCs isolated from carotid and femoral artery of *Apoe−/−* mice using immunofluorescence staining via smooth muscle Actin (SMA, green) and co‐staining with smooth muscle myosin heavy chain (SMMHC, red). Scale bar = 20 μm. (B) Quantification of calcium content/protein ratio in carotid (Car.) and femoral (Fem.) vascular smooth muscle cells (VSMCs) under normal media (NM) or calcifying media (CM) for 7 days, *n* = 5–7 per group (C) Gene expression of *Bmp2* and *Runx2* in carotid and femoral VSMCs after 7‐day incubation with normal or calcifying media via digital droplet PCR, *n* = 7–14 per group. (D, E) Immunofluorescent imaging of (D) BMP2, (E) TNAP and (F) RUNX2 (nuclear associated) in carotid and femoral VSMCs after 7‐day incubation with normal or calcifying media. Scale bar = 20 μm, *n* = 5 per group. Each dot/point on the graph represents an independent sample. Bar graphs represent mean ± SEM. Mann–Whitney test was used for two‐group comparisons, and ordinary one‐way ANOVA followed by Tukey's multiple comparisons post test was used for multiple group comparisons. *p* values are depicted, with values ≤ 0.05 considered significant.

To investigate calcification‐specific differences between carotid and femoral arteries identified in vivo in more mechanistic detail, we next isolated and characterized carotid and femoral VSMCs from *Apoe−/−* mice. Immunofluorescence staining and bright field imaging for alpha‐smooth muscle Actin (α‐SMA) and co‐staining with smooth muscle myosin heavy chain (SMMHC) confirmed colocalization of both markers in the isolated cells, verifying their identity as VSMCs (Figure 2A, Figure S2A). Furthermore, we evaluated the cellular viability of carotid and femoral VSMCs in a 7‐day culture with NM or CM. Here, neither an Alamar blue assay (Figure S2B) nor immunofluorescence staining with Annexin V (Figure S2C) revealed differences in VSMC viability in different culture media. In line, treatments with DDR1 stimulator, inhibitor, and NF‐κB inhibitor did not impact cell viability during NM and CM conditions (Figure S2D).

Next, we measured the ratio of calcium content to protein levels in carotid and femoral VSMCs cultured for 7 days in either NM or CM. Under NM conditions, no significant differences in calcium content were observed between carotid and femoral VSMCs (Figure 2B). In contrast, after 7 days in CM, femoral VSMCs displayed significantly higher levels of calcification compared to carotid VSMCs (Figure 2B). To further investigate this on a molecular level, we analyzed mRNA expression of calcification‐related proteins under both conditions using digital droplet PCR, which revealed increased expression of *Bmp2* and *Runx2* in femoral VSMCs exposed to CM for 7 days (Figure 2C). Consistently, immunofluorescence staining demonstrated greater prevalence of *BMP2, TNAP*, and *RUNX2* in femoral VSMCs compared with carotid VSMCs under calcifying conditions (Figure 2D–F). In addition, we cultured arterial rings harvested from *Apoe−/−* donor mice in NM or CM and detected an increased calcium to protein ratio in femoral arterial rings incubated with CM for 7 days (Figure S2E).

Conclusively, these cell culture results clearly confirm the in vivo findings, demonstrating an increased calcification propensity and an increased prevalence and expression of the differentially expressed mediators of calcification in femoral compared to carotid vascular beds.

### 3.3 Role for DDR1 in Calcification of Femoral but Not Carotid VSMCs

DDR1 was previously shown to sense arterial stiffness and to form a mechanistic link between stiffness and calcification [22]. Thus, considering the role of arterial stiffness in the progression of LEAD, we further investigated the potential role of DDR1 in the calcification of the femoral arteries.

**FIGURE 3:**
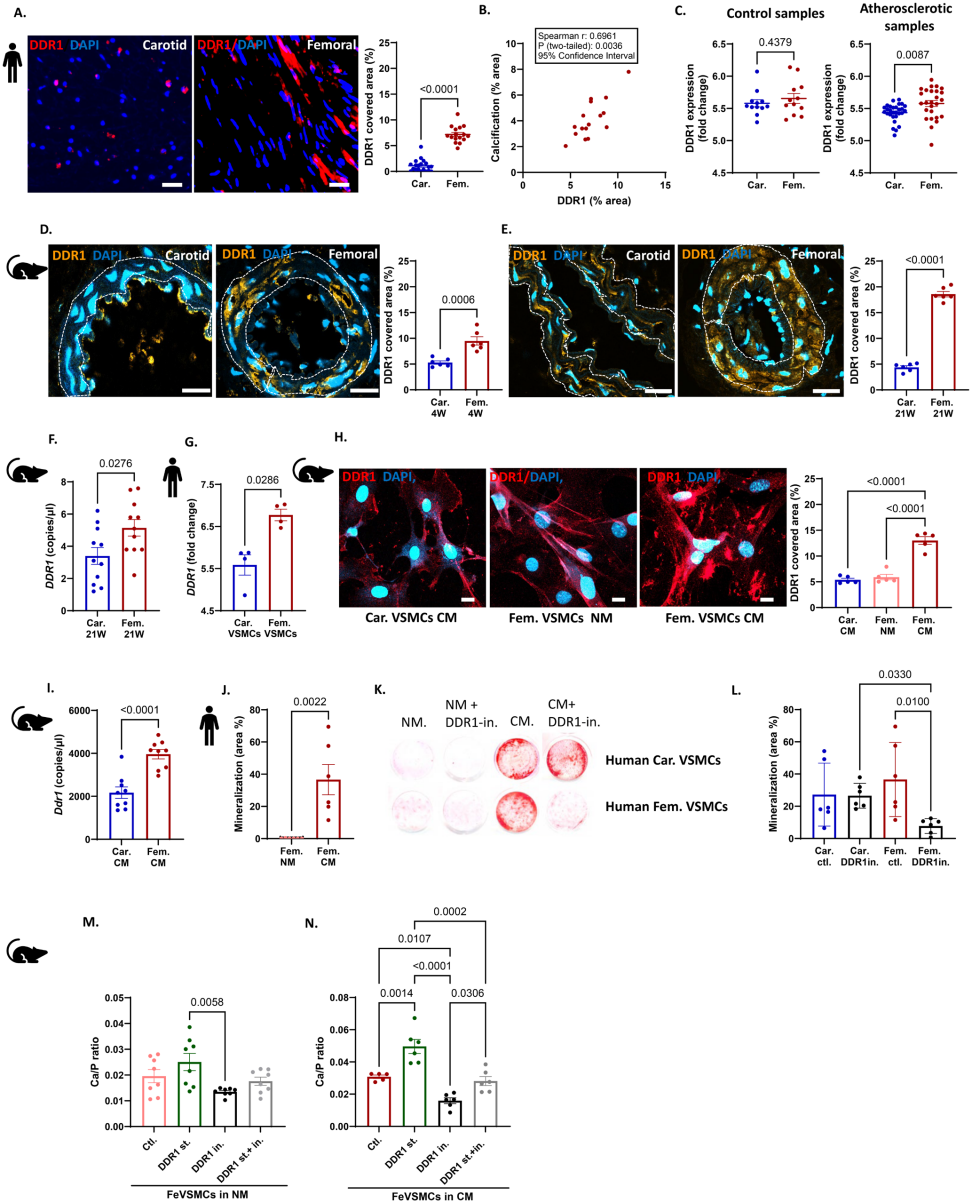
Role of DDR1 in medial arterial calcification of femoral and carotid arteries and VSMCs. (A) Immunofluorescence staining and quantification of DDR1 in carotid and femoral endarterectomies from atherosclerotic patients. Scale bar = 100 μm, *n* = 20 per sample. (B) Correlation between DDR1+ area and calcified area in the femoral artery obtained from atherosclerotic patients, *n* = 16. (C) Quantification of DDR1 gene expression (fold change) in non‐atherosclerotic and atherosclerotic human carotid and femoral artery samples, *n* = 12–22 samples per group. (D, E) Immunofluorescent imaging and quantification of DDR1+ area in the medial layer of carotid and femoral arteries of *Apoe−/−* fed with 4 weeks (D) and 21 weeks (E) of western diet. Scale bar = 20 μm, *n* = 6 per group. (F) *Ddr1* expression (copies/μl) in carotid and femoral arteries of *Apoe−/−* mice on 21w of western diet using digital droplet PCR, *n* = 10 per group. (G) Gene expression of *DDR1* in human carotid and femoral VSMCs incubated in calcifying medium for 7 days. (H) Immunofluorescent imaging of DDR1 in carotid and femoral VSMCs after 7‐day incubation with normal (NM) or calcifying media (CM). Scale Bar = 20 μm, *n* = 4 per group. (I) Gene expression of *Ddr1* using digital droplet PCR in murine carotid and femoral VSMCs incubated in calcifying medium for 7 days (*n* = 7 per group). (J) Quantification of mineralization in cultured human femoral VSMCs incubated in calcifying media for 7 days, *n* = 6 per group. (K) Representative pictures of Alizarin red staining on human carotid and femoral VSMCs incubated in normal or calcifying condition for 7 days. (L) Quantification of mineralization in human carotid and femoral VSMCs in the presence of DDR1 inhibitor DDR1‐IN‐1 (DDR1 in.), *n* = 6 per group. (M, N) Ratio analyses of calcium content and protein after 7‐day incubation with (M) normal media or (N) calcifying media with DDR1 stimulator (DDR1st.) or DDR1 inhibitor (DDR1in.) or with DDR1 stimulator and inhibitor (DDR1 st. + in.), *n* = 6 per group. Bar graphs represent mean ± SEM, each dot and n represent individual animal or experiment number. Unpaired *t*‐test, Mann–Whitney test, nonparametric Spearman correlation, Kruskal–Wallis tests and ordinary one‐way ANOVA followed by Tukey's multiple comparisons post test was used for multiple group comparisons.

Immunofluorescence staining of patient samples revealed a higher prevalence of DDR1 in femoral compared to carotid atherosclerotic patient arterial specimens (Figure 3A). Moreover, a positive correlation was observed between calcification levels and DDR1 expression in femoral arteries (Figure 3B). Re‐analysis of open‐access human gene expression data (GSE100927) [18] showed significantly higher DDR1 expression in atherosclerotic femoral specimens compared to carotid specimens. Such a difference was not observed in non‐atherosclerotic human donors (Figure 3C), again suggesting an important inflammatory component as a contributor.

Similar findings were obtained in murine models, where femoral arteries of *Apoe−/−* mice fed a WD for 4 or 21 weeks exhibited greater DDR1 positivity compared to carotid arteries (Figure 3D,E). Femoral arteries of *Apoe−/−* mice on a 21‐week WD also showed significantly elevated *Ddr1* gene expression (Figure 3F).

To further validate these findings in cell culture setups in an independent cohort, we re‐analyzed open‐access gene expression data from human carotid and femoral VSMCs (GSE84012) [19] incubated in CM. VSMCs from the lower extremity (femoral and popliteal artery) showed significantly higher *DDR1* expression when compared to thoracic arteries (carotid and aorta) (Figure 3G). In line, in murine cell culture experiments, immunofluorescent staining demonstrated a higher DDR1+ area in femoral VSMCs cultured in CM compared to carotid VSMCs in CM or femoral VSMCs in NM (Figure 3H). Furthermore, mRNA analysis confirmed significantly higher *Ddr1* expression in murine femoral VSMCs under calcifying conditions (Figure 3I).

Next, we evaluated the effects of DDR1 modulation on calcification using cell culture experiments with DDR1 inhibitors and stimulators in human and murine VSMCs. We first used different concentrations of the DDR1 inhibitor DDR1‐IN‐1 to assess its role in calcification, indicating a significant downregulation at 1 μM when compared to CM control, and this without inducing cellular apoptosis (Figure S3A). Thereafter, we performed ddPCR to evaluate the effect of DDR1 stimulation and inhibition on *Ddr1* expression. Results show substantial stimulation of Ddr1 expression upon DDR1 stimulation versus a significant reduction upon DDR1 inhibition (Figure S3B). Similar findings were observed via immunofluorescence staining (Figure S3C). The effect of DDR1 stimulator and inhibitor was not observed for DDR2 (Figure S3D). CM induced substantial mineralization in human femoral VSMCs (Figure 3J) while the addition of the DDR1 inhibitor (DDR1‐IN‐1) significantly reduced mineralization (Figure 3K,L). Such DDR1‐mediated calcification reduction was not observed in human carotid VSMCs under calcifying conditions (Figure 3K,L). Similar effects of DDR1 inhibition were observed in murine VSMCs, as shown via Alizarin Red staining (Figure S3H). No significant changes were observed with DDR1 stimulation or inhibition vs. control in VSMCs cultured in NM (Figure 3M). However, murine femoral VSMCs exhibited increased calcium content when treated with a DDR1 stimulator (collagen type‐1), while DDR1 inhibitor (DDR1‐IN‐1) significantly reduced calcification of murine femoral VSMCs (Figure 3N). Together, these results demonstrate a crucial role for DDR1 in promoting calcification of femoral, but not carotid, VSMCs, highlighting a vascular site‐specific DDR1 involvement in vascular calcification.

### 3.4 DDR1 Reduces Medial Arterial Calcification via Activation of NF‐κB p65

**FIGURE 4:**
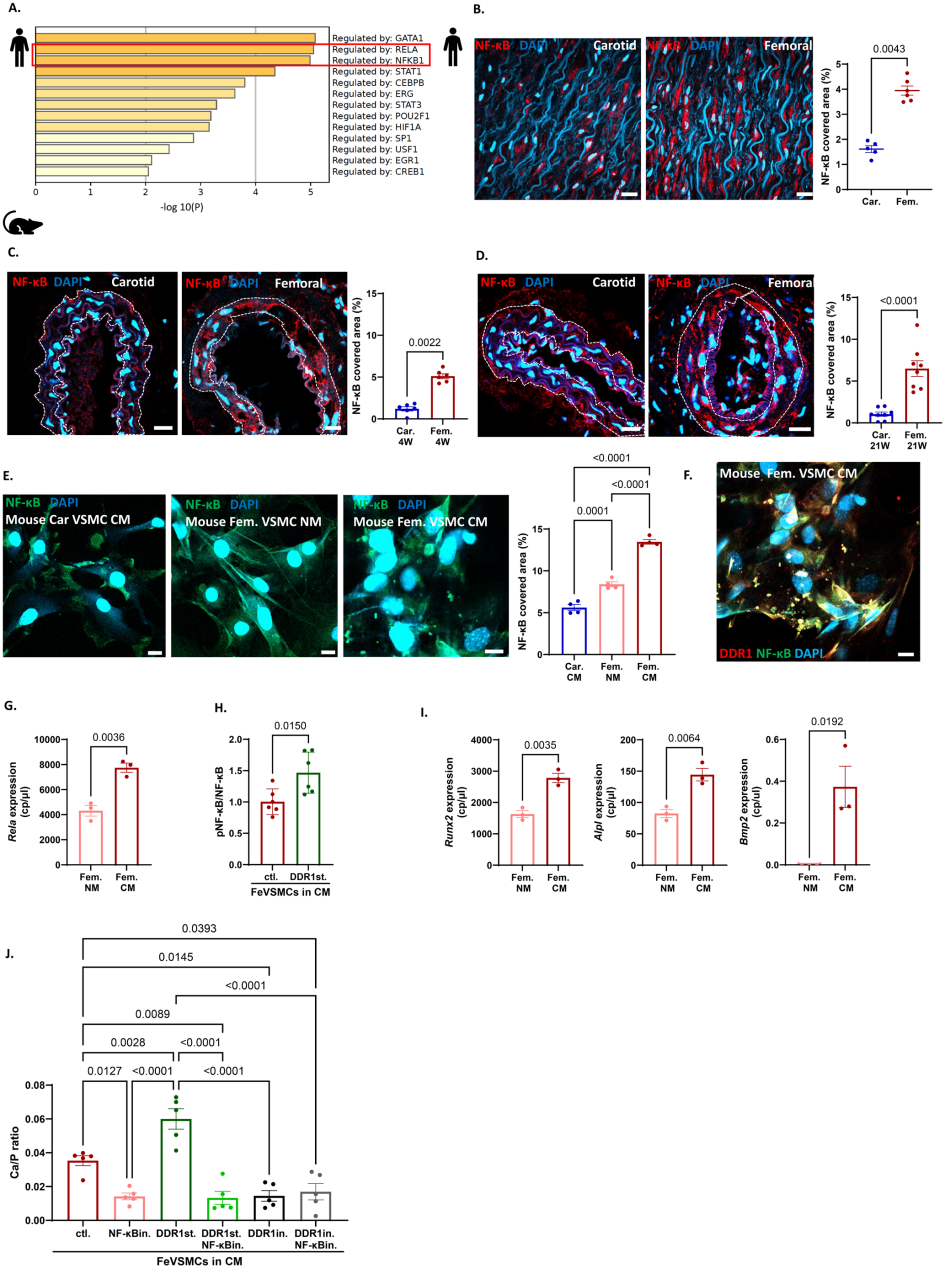
DDR1 reduces medial arterial calcification via activation of NF‐κB p65. (A) Analyses of potential transcription factors regulating differential gene expression in carotid vs. femoral endarterectomies using the GSE100927 data set in TTRUST, identifying NF‐κB as differently regulated. (B) Quantification of NF‐κB covered area in the medial layer of atherosclerotic carotid and femoral artery sample from patients using immunofluorescence, Scale bar = 100 μm, *n* = 5–6 per group. (C, D) Immunofluorescent imaging and quantification of NF‐κB in carotid and femoral arteries of *Apoe−/−* fed with 4 weeks (C) and 21 weeks (D) of western diet. Scale Bar = 20 μm, *n* = 6–8 per group. (E) Immunofluorescent staining and quantification of NF‐κB in carotid and femoral VSMCs under normal and calcifying conditions for 7 days. (F) Co‐staining of NF‐κB (green) with DDR1 (red) in femoral VSMCs incubated in calcifying media for days. Scale Bar = 20 μm, *n* = 4 per group. (G) Gene expression of *Rela* using digital droplet PCR in murine carotid and femoral VSMCs incubated in normal or calcifying medium for 7 days, *n* = 3 per group. (H) Quantification of ratio of phosphorylated NF‐κB to total NF‐κB during stimulation of DDR1 in the femoral VSMCs in calcifying media using western blot, *n* = 6 per group. (I) Quantification of gene expression of *Runx2*, *Bmp2* and *Alpl* in femoral VSMCs incubated in CM for 7 days, *n* = 3 per group. (J) Quantification of calcium to protein ratio in the presence of NF‐κB inhibitor (NF‐κBin. = MG132) incubated in calcifying media with DDR1 stimulator (DDr1st.) or with DDR1 inhibitor (DDR1in.), *n* = 5 per group. Bar graphs represent mean ± SEM. Each dot/point on the graph represents an independent sample. Mann–Whitney test, Kruskal–Wallis test and ordinary one‐way ANOVA followed by Tukey's multiple comparisons post test was used for multiple group comparisons.

To identify pathways relevant to femoral artery calcification, we analyzed the GSE100927 dataset using the TTRUST database. This analysis highlighted an increased gene regulation by transcription factors RELA (NF‐κB p65) and NF‐κB1 (NF‐κB p50) in human atherosclerotic femoral samples as compared to carotid samples (Figure 4A), with p65/p50 the prototypical dimer driving NF‐κB‐dependent gene expression and NF‐κB known to regulate vascular calcification [16]. Hence, we evaluated NF‐κB p65 prevalence in human carotid vs. femoral artery samples. Immunofluorescence analysis revealed an increase in NF‐κB p65‐covered area in femoral patient samples compared to carotid patient specimens (Figure 4B). Similarly, *Apoe−/−* mice fed a WD for 4 or 21 weeks showed a higher NF‐κB‐covered area in femoral arteries compared to carotid arteries (Figure 4C,D).

To further investigate these findings in VSMCs in vitro, murine carotid and femoral VSMCs were cultured in NM or CM. NF‐κB p65 prevalence was significantly higher in femoral VSMCs incubated with CM compared to carotid VSMCs in CM or femoral VSMCs in NM (Figure 4E). Colocalization studies using immunofluorescence staining demonstrated DDR1 co‐localizing with NF‐κB p65 in CM‐treated femoral VSMCs (Figure 4F).

To further investigate *Rela* expression and the effect of DDR1 stimulation on NF‐κB p65 (*Rela* expression), we specifically analyzed femoral VSMCs (FeVSMCs) in NM and CM. Firstly, *Rela* expression was significantly increased in FeVSMCs under calcifying conditions using CM (Figure 4G). Additionally, western blot analysis confirmed increased phosphorylated NF‐κB levels in femoral VSMCs upon DDR1 stimulation, indicating DDR1‐mediated activation of the NF‐κB pathway (Figure 4H, Figure S3G). Furthermore, we observed higher expression of calcifying genes (*Runx2*, *Alpl*, and *Bmp2*) in the same FeVSMCs incubated in CM, suggesting a connection between *Rela* expression and pro‐calcifying gene expression (Figure 4I). To further investigate the effect of NF‐κB inhibition on DDR1‐induced calcification in femoral VSMCs, we performed calcification analyses in the presence of an NF‐κB inhibitor with or without the addition of DDR1 stimulator or inhibitor. First, we evaluated the efficiency of the NF‐κB inhibitor using an ELISA for total and phosphorylated (active) NF‐κB p65, showing no significant changes in total NF‐κB p65 (Figure S3E) but a significant reduction in phosphorylated (active) NF‐κB p65 upon NF‐κB inhibition (Figure S3F). Furthermore, our results showed a significant increase in calcification upon DDR1 activation, which was significantly reduced upon addition of the NF‐κB inhibitor MG123. In contrast, in the presence of a DDR1 inhibitor, NF‐κB inhibition did not further reduce calcification, underlining the importance of DDR1 in stimulating NF‐κB‐dependent calcification (Figure 4J). Altogether, these analyses suggest the involvement and relationship between DDR1‐ and NF‐κB‐induced upregulation of calcification genes and calcium content in femoral VSMCs in calcifying conditions.

## 4 Discussion

This study investigated calcification‐related differences between femoral and carotid arteries using patient samples, an atherosclerotic mouse model, and isolated VSMCs, revealing a stronger calcification propensity in femoral arterial beds. Furthermore, we identified DDR1 as a key regulator of medial calcification, specifically in femoral arteries during inflammation, and identified a DDR1‐induced upregulation of calcifying gene expression in femoral VSMCs via the activation of NF‐κB.

Calcification analyses of femoral and carotid arteries obtained from human patient samples exhibited a greater propensity for calcification in femoral TE samples as compared to carotid TE samples. Similar findings were observed in the medial layer of femoral arteries from *Apoe−/−* mice fed a WD for 4 and 21 weeks. These animals were used as a model to induce hyperlipidemia and pro‐inflammatory conditions, which are shown to induce phenotypic switching of VSMCs to an osteogenic phenotype, thereby leading to MAC [17]. Switched VSMCs produce pro‐inflammatory cytokines, which can further exacerbate vascular stiffness [23]. Our findings also align with previous studies, which reported significantly higher calcification levels in both atherosclerotic and healthy femoral arteries relative to other vascular regions [1, 18]. Furthermore, prior research has identified fibrous cap atheroma as a characteristic feature of carotid arteries, whereas fibrocalcific plaques and osteoid metaplasia are more commonly associated with femoral arteries [24]. Additionally, MAC is highly prevalent in LEAD, being far more prominent than in any other arterial bed and strongly associated with an increased risk of limb amputation [6]. This was further confirmed in our calcification analyses in the medial layer of femoral arteries from *Apoe−/−* mice fed a WD for 4 and 21 weeks. In our in vitro experiments using both mouse and human VSMCs isolated from femoral and carotid arteries, femoral VSMCs were significantly more prone to calcification during calcifying conditions compared to carotid VSMCs, aligning with earlier studies in human cells [19]. In parallel with enhanced calcification, we observed an increase in calcification‐regulating proteins (RUNX2, BMP‐2, TNAP), especially in femoral arteries. However, we have also detected certain discrepancies between RUNX2 protein and *Runx2* mRNA expression, which may reflect an impact of posttranscriptional and posttranslational regulation. RUNX2 is known to be stabilized via phosphorylation and protected from proteasomal degradation under certain signaling contexts. Such translational regulation or reduced turnover may explain the elevated protein levels in the absence of transcriptional upregulation. Collectively, these findings emphasize that femoral arteries and femoral VSMCs are inherently more susceptible to calcification.

Arterial stiffness is one of the hallmarks of LEAD [25] and may be influenced by DDR1, which has been previously suggested to act as a mechano‐sensor of matrix stiffness in aortic VSMCs [22]. Additionally, DDR1 has been shown to regulate calcification‐related genes in osteoblasts, where its knockout led to increased cortical bone thickness and a downregulation of key osteogenic markers, including RUNX2, BMP2, and ALP [14]. In a different study, DDR1 activation was reported to drive a stiffness‐dependent increase of vascular calcification in aortic VSMCs [22]. When we analyzed previously available gene expression data from human aorta/carotid and femoral/popliteal arteries, it showed a significant upregulation of DDR1 in the arteries of lower extremities compared to carotid and thoracic aorta. These findings support the notion that DDR1 may play a more important role in the calcification of femoral compared to the carotid artery. Along these lines, our data also demonstrated a significantly higher expression of DDR1 in femoral arteries and femoral VSMCs compared to the carotid vascular bed, both for human and murine origin, under inflammatory and calcifying conditions. In line, DDR1 inhibition had no effect on the calcification of carotid VSMCs, while inhibition of DDR1 specifically reduced calcification and mineralization in both human and murine femoral VSMCs. This highlights a vascular bed‐specific and important role of DDR1 in femoral artery calcification.

Only a limited number of studies have explored DDR1‐induced arterial calcification using aortic models [22, 26], and knowledge regarding DDR1‐mediated medial calcification in femoral arteries remained elusive so far. Given that femoral arteries are inherently more susceptible to calcification [1], and our observed higher prevalence of DDR1 in femoral arteries under inflammatory and calcifying conditions, we further investigated the mechanisms underlying DDR1‐induced femoral calcification in LEAD.

Previous studies have shown that DDR1‐mediated intimal vascular calcification in the aorta is mediated via phosphoinositide 3‐kinase/Akt signaling [26]. Transcriptomic analysis of atherosclerotic patient femoral thromboendarterectomy samples established TGFβ signaling to be specifically upregulated in femoral arteries [19]. Furthermore, DDR1 also activates the nuclear factor‐kappa B (NF‐κB) pathway [27], which has been involved in the upregulation of BMP2 [28]. Nonetheless, DDR1‐induced signaling pathways in medial femoral calcification have not been explored. Reanalyzing the open GSE100927 dataset with the TTRUST database showed an upregulation of NF‐κB p65 (RELA) in human femoral endarterectomy samples when compared to carotid endarterectomies. Furthermore, our data on atherosclerotic patient endarterectomies and atherosclerotic animals showed increased NF‐κB p65 prevalence in the femoral arteries. However, so far, there have been no studies suggesting a role for a DDR1—NF‐κB axis in inducing femoral MAC. Here, we show for the first time in cell culture experiments that DDR1 stimulation triggers a substantial increase in activated NF‐κB in femoral VSMCs. These data are further supported by immunofluorescence staining of femoral VSMCs in CM, showing colocalization and association of DDR1 with NF‐κB. Furthermore, DDR1‐induced calcification in femoral VSMCs was completely blocked by NF‐κB inhibition. Together, these results position DDR1 as a central regulator of femoral medial calcification and suggest that pharmacological targeting of DDR1 could represent a novel therapeutic strategy for preventing MAC in LEAD.

In conclusion, in this study, we have illustrated vascular bed‐specific differences in calcification: femoral arteries are more prone to MAC, which is linked to a stronger upregulation of DDR1 specifically in femoral arteries in conditions of calcification. We identified DDR1 as a key player driving femoral calcification in LEAD via the activation of the transcription factor NF‐κB, the latter driving the expression of the calcifying proteins BMP2 and RUNX2.

### 4.1 Limitations and Prospects

One of the main limitations of this study is the limited availability of human tissue from our collaborating surgical units, where carotid endarterectomy specimens are more routinely collected as compared to the femoral endarterectomy specimens. While the numbers of included carotid and femoral arteries are therefore unequal, we ensured that all samples used met the same inclusion criteria and were processed using identical protocols. It was particularly difficult to interpret any sex‐specific differences in femoral arterial calcification due to the small sample size in femoral specimens. Furthermore, we analyzed data from open‐access databases, but detailed information on the exact nature of the “control” samples (whether being arteries without plaque from diseased patients or truly healthy tissues from non‐atherosclerotic donors) was not available. This limits our ability to fully interpret the lack of difference in for example, *DDR1* gene expression between control and atherosclerotic vessel specimens within the same vascular bed. Other limitations include the broader effects of the applied NF‐κB inhibitor MG132 in this study and the need for long‐term and systemic evaluation of DDR1‐targeting strategies in vivo, including the comparison of knockout vs. inhibition. We have furthermore observed certain differences between the in vitro, in vivo and human models especially regarding the regulation of calcifying gene expressions, although all pointing overall to the same conclusion of increased calcification mechanisms in femoral compared to carotid artery. Also, while we focused our analysis on medial regions to investigate vessel wall‐intrinsic differences between carotid and femoral beds, we recognize that calcification may vary across different layers and regions of the arterial wall. For instance, areas under atherosclerotic plaques may exhibit enhanced VSMC osteogenic transdifferentiation due to local inflammatory and oxidative stimuli. Future spatially resolved analyses (spatial transcriptomics) could help clarify microenvironmental effects on calcification phenotypes.

## Author Contributions

M.T. carried out experiments, analyzed data, and drafted figures; T.Q., N.A., M.S., Y.J., Y.Y., and J.W. contributed to in vitro and in vivo experiments and data analysis; C.G., L.M., N.S., M.S., H.N., and D.K. provided critical review of the manuscript; Y.D. provided infrastructure and funding and supervised the study and planned experiments; M.T. and Y.D. reviewed data, wrote, and revised the manuscript. All authors have read and contributed to the article and approved submission.

## Funding

This work was supported by the Deutsche Forschungsgemeinschaft (DFG) SFB1123‐A1, SFB/TRR219‐ID322900939‐(project‐C02‐to‐CG‐and‐M‐05‐to‐HN) and the Swiss National Science Foundation Project IDs 310030_197655 as well as the Novartis foundation (grant number 22B148) to Y.D and Jubiläumsstiftung von Swiss Life to M.T. This work was also supported by the Deutsche Forschungsgemeinschaft (German Research Foundation) grant GO1804/9–1. Furthermore, this study was supported by the Project of National Natural Science Foundation of China (grant number 82200065) to Y.Y and the Fondation de l'Avenir (Paris, France), the University Hospital of Nantes (Nantes, France), and the Societé de chirurgie vasculaire et endovasculaire (Paris, France) to T.Q.

## Conflicts of Interest

The authors declare no conflicts of interest.

## Supporting information


**Figure S1:** (A) Quantification of *BMP2*, *ALPL*, and *RUNX2* gene expression (fold change) in healthy carotid and femoral artery samples from dataset GSE100927. (B) Expression of *Runx2*, *Bmp2*, and *Alpl* in fold change (normalized to *Gapdh*) in carotid and femoral arteries of *Apoe−/−* mice on 4 weeks of western diet (WD), *n* = 3–6 per sample. (C) Immunofluorescent imaging and quantification of tissue nonspecific alkaline phosphatase (TNAP) in the medial layer of carotid and femoral arteries of *Apoe−/−* fed with 4 weeks and (D) 21 weeks of WD, *n* = 6 per group. Dot plots (for Human samples) and Bar graphs (for murine samples) represent mean ± SEM. Each dot/point on the graph represents an independent sample. Mann–Whitney test is used to compare the groups.
**Figure S2:** (A) Bright field imaging of cultured VSMCs isolated from carotid and femoral artery of *Apoe−/−* mice. Scale bar = 100 μm. (B) Analyses of cellular viability by measuring absorbance (570–600 nm) through the reduction of Alamar Blue reagent in murine carotid and femoral VSMCs incubated for 7 days in normal media (NM) or calcifying media (CM), *n* = 6 per group. (C) Immunofluorescent imaging of annexin V in murine carotid and femoral VSMCs after 7‐day incubation with NM or CM. Scale Bar = 20 μm. (D) Analyses of cellular viability was done by measuring absorbance (570–600 nm) through the reduction of Alamar Blue reagent in murine carotid and femoral VSMCs incubated for 7 days in NM or CM with DDR1 stimulator (collagen type‐1, 10 μg/mL), DDR1 inhibitor (DDR1‐IN‐1, 1 μM) and NF‐κB‐p65 (MG132, 1 ng/mL), *n* = 6–8 per group. (E) Calcium/protein ratio in murine carotid or femoral arterial rings incubated in NM or CM for 7 days, *n* = 3 per group. Bar graphs represent mean ± SEM. Each dot/point on the graph represents an independent sample. Mann–Whitney test and ordinary one‐way analysis of variance (ANOVA) followed by Tukey's multiple comparisons post test were used for multiple group comparisons.
**Figure S3:** (A) Calcium/protein ratio in murine femoral VSMCs incubated in CM for 7 days with different concentrations of DDR1 inhibitor, *n* = 3 per group. (B) *Ddr1* gene expression & (C) Immunofluorescence staining in murine femoral VSMCs incubated in CM for 7 days with DDR1 stimulator and inhibitor. (D) Immunofluorescent imaging of DDR2 in murine FeVSMCs during incubation in CM with DDR1 stimulator and inhibitor for 7 days. (E) ELISA analyses of total NF‐κB p65 and (F) Phosphorylated NF‐κB p65 in murine femoral VSMCs incubated in CM with and without NF‐κB inhibitor, *n* = 6 per group. (G) Western blots used during analyses of pNF‐κB/total NF‐κB ratio during stimulation of DDR1 in the murine femoral VSMCs in CM (with quantification in Figure 4H). (H) Alizarin red staining of murine femoral VSMCs in NM and CM with DDR1 inhibitor. Each dot/point on the graph represents an independent sample. Mann–Whitney test and ordinary one‐way ANOVA followed by Tukey's multiple comparisons post test was used for multiple group comparisons.
**Table S1:** Tabular representation of clinical and demographic information of the human subjects from whom carotid and femoral artery samples were obtained summarizes age, sex distribution, hypertension, diabetes, smoking status, Dyslipidemia, chronic heart disease, renal function (dialysis), ASA classification of the patient's physical status, BMI, and statin use.

## Data Availability

The data that support the findings of this study are available from the corresponding author upon reasonable request.
