# Supplementary material for: DDR1 Regulates Femoral Arterial Calcification in Lower‐Extremity Artery Disease Through NF‐Kappa B Activation
Source: Acta Physiol (Oxf). 2025 Dec 16;242(1):e70146. doi: 10.1111/apha.70146 (PMC12706703; doi:10.1111/apha.70146)
Supplement: Supplementary file 1 — Figure S1: (A) Quantification of BMP2, ALPL, and RUNX2 gene expression (fold change) in healthy carotid and femoral artery samples from dataset GSE100927. (B) Expression of Runx2, Bmp2, and Alpl in fold change (normalized to Gapdh) in carotid and femoral arteries of Apoe−/− mice on 4 weeks of western diet (WD), n = 3–6 per sample. (C) Immunofluorescent imaging and quantification of tissue nonspecific alkaline phosphatase (TNAP) in the medial layer of carotid and femoral arteries of Apoe−/− fed with 4 weeks and (D) 21 weeks of WD, n = 6 per group. Dot plots (for Human samples) and Bar graphs (for murine samples) represent mean ± SEM. Each dot/point on the graph represents an independent sample. Mann–Whitney test is used to compare the groups. Figure S2: (A) Bright field imaging of cultured VSMCs isolated from carotid and femoral artery of Apoe−/− mice. Scale bar = 100 μm. (B) Analyses of cellular viability by measuring absorbance (570–600 nm) through the reduction of Alamar Blue reagent in murine carotid and femoral VSMCs incubated for 7 days in normal media (NM) or calcifying media (CM), n = 6 per group. (C) Immunofluorescent imaging of annexin V in murine carotid and femoral VSMCs after 7‐day incubation with NM or CM. Scale Bar = 20 μm. (D) Analyses of cellular viability was done by measuring absorbance (570–600 nm) through the reduction of Alamar Blue reagent in murine carotid and femoral VSMCs incubated for 7 days in NM or CM with DDR1 stimulator (collagen type‐1, 10 μg/mL), DDR1 inhibitor (DDR1‐IN‐1, 1 μM) and NF‐κB‐p65 (MG132, 1 ng/mL), n = 6–8 per group. (E) Calcium/protein ratio in murine carotid or femoral arterial rings incubated in NM or CM for 7 days, n = 3 per group. Bar graphs represent mean ± SEM. Each dot/point on the graph represents an independent sample. Mann–Whitney test and ordinary one‐way analysis of variance (ANOVA) followed by Tukey's multiple comparisons post test were used for multiple group comparisons. Figure S3: (A) Calcium/protein ratio [file APHA-242-e70146-s001.pdf]

Sup. Figure 1.

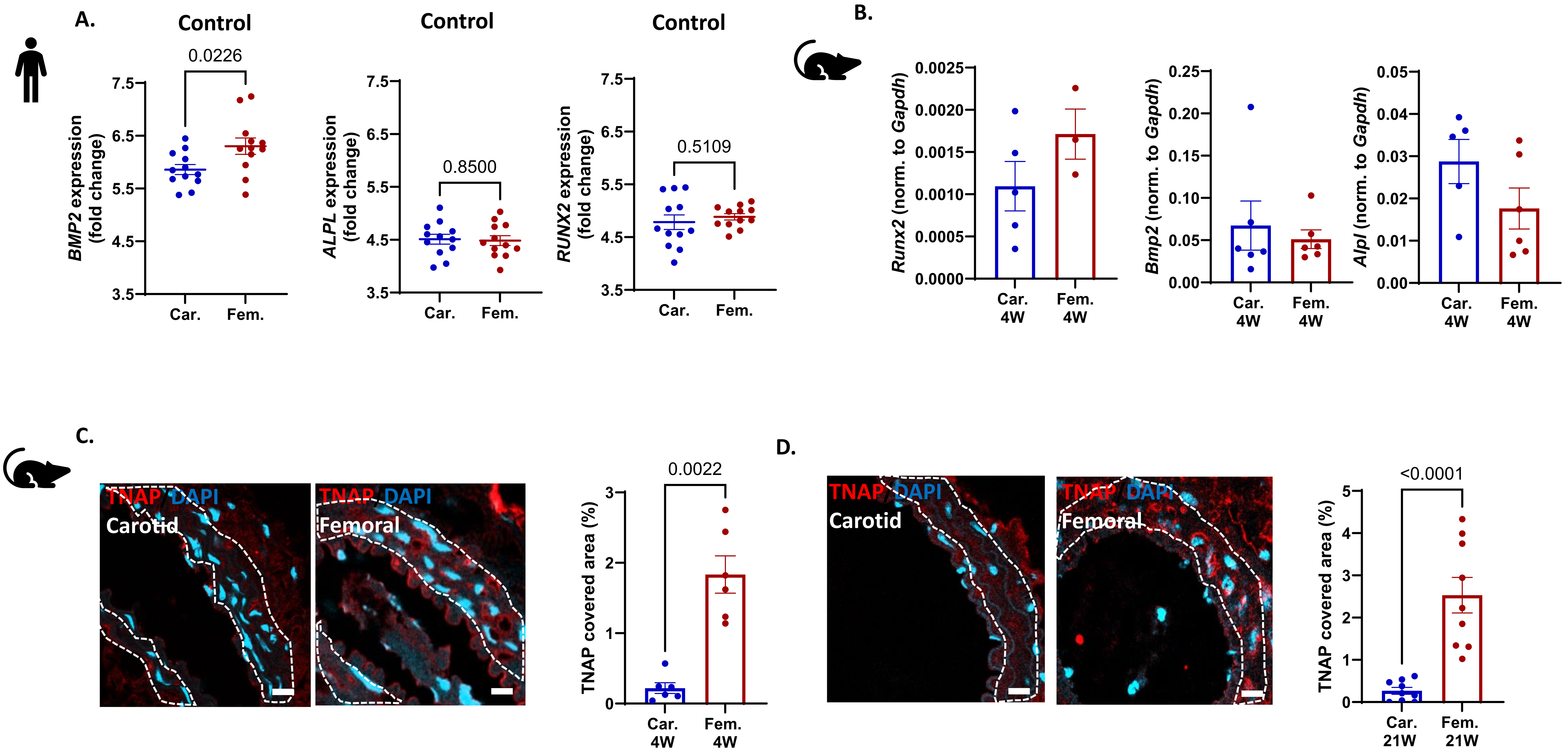

A.
Control
0.0226
BMP2 expression (fold change)
Car.
Fem.
Control
0.8500
ALPL expression (fold change)
Control
0.5109
RUNX2 expression (fold change)
B.
Runx2 (norm. to Gapdh)
Bmp2 (norm. to Gapdh)
Alpl (norm. to Gapdh)
Car. 4W
Fem. 4W
C.
TNAP DAPI
Carotid
Femoral
0.0022
TNAP covered area (%)
D.
<0.0001
Car. 21W
Fem. 21W

Sup. Figure 2.

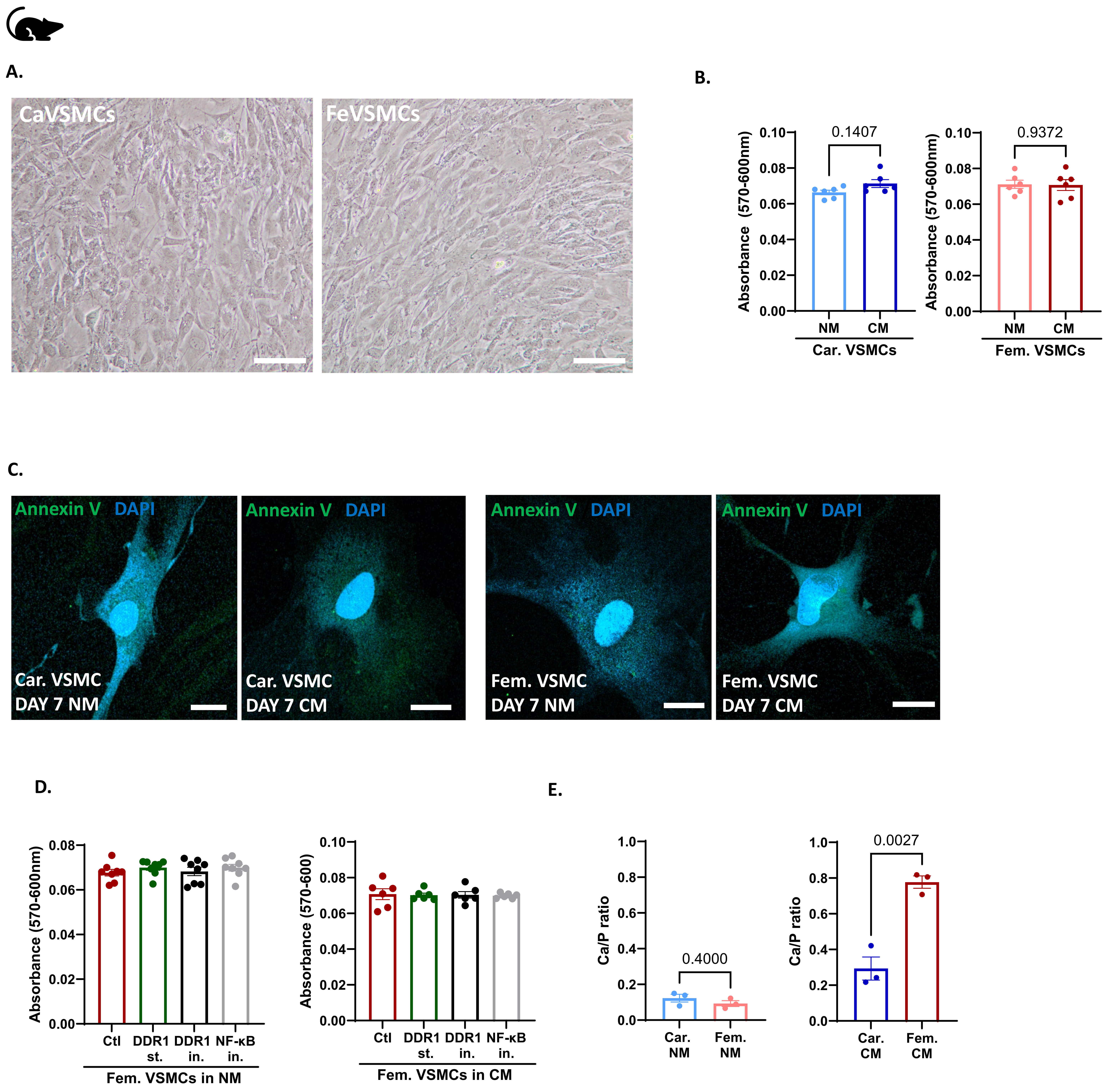

A.
CaVSMCs
FeVSMCs
B.
Absorbance (570-600nm)
0.10
0.08
0.06
0.04
0.02
0.00
0.1407
NM
CM
Car. VSMCs
0.9372
NM
CM
Fem. VSMCs
C.
Annexin V DAPI
Car. VSMC DAY 7 NM
Car. VSMC DAY 7 CM
Fem. VSMC DAY 7 NM
Fem. VSMC DAY 7 CM
D.
Absorbance (570-600nm)
Absorbance (570-600)
Ctl
DDR1 st.
DDR1 in.
NF-κB in.
Fem. VSMCs in NM
Fem. VSMCs in CM
E.
Ca/P ratio
1.0
0.8
0.6
0.4
0.2
0.0
0.4000
Car. NM
Fem. NM
0.0027
Car. CM
Fem. CM

Sup. Figure 3.

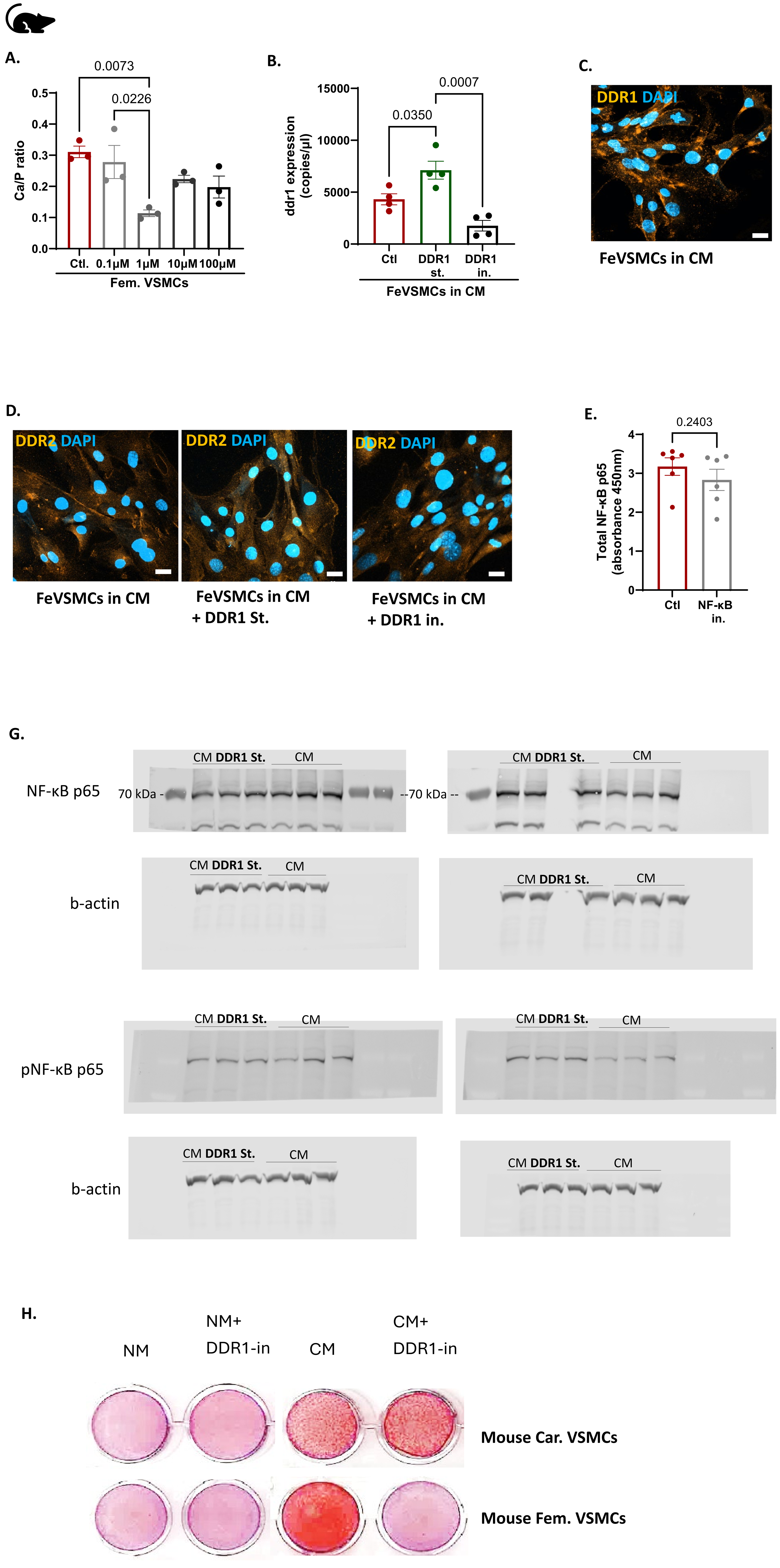
A.
0.0073
0.0226
Ca/P ratio
0.5
0.4
0.3
0.2
0.1
0.0
Ctl.
0.1µM
1µM
10µM
100µM
Fem. VSMCs
B.
15000
10000
5000
0
ddr1 expression (copies/µl)
0.0007
0.0350
Ctl
DDR1 st.
DDR1 in.
FeVSMCs in CM
C.
DDR1 DAPI
FeVSMCs in CM
FeVSMCs in CM + DDR1 St.
FeVSMCs in CM + DDR1 in.
D.
DDR2 DAPI
FeVSMCs in CM
FeVSMCs in CM + DDR1 St.
FeVSMCs in CM + DDR1 in.
E.
0.2403
Total NF-κB p65 (absorbance 450nm)
4
3
2
1
0
Ctl
NF-κB in.
F.
0.0216
pNF-κB p65 (absorbance 450nm)
0.10
0.08
0.06
0.04
0.02
0.00
Ctl
NF-κB in.
G.
NF-κB p65
70 kDa
CM DDR1 St.
CM
b-actin
pNF-κB p65
H.
NM
NM+ DDR1-in
CM
CM+ DDR1-in
Mouse Car. VSMCs
Mouse Fem. VSMCs

Sup. Table 1.

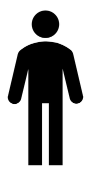

Patient Samples	Age	Sex	Hypertension	Diabetes	Smoking	Dyslipidemia	CHD	Dialysis	ASA Classification	Size (cm)	Weight (kg)	BMI	Statin
C1	73	male	yes	no	ex	no	yes	no	3	171	62	21.2	yes
C2	84	male	yes	no	ex	yes	no	no	3	163	58	21.8	yes
C3	69	male	yes	yes	ex	yes	no	no	3	173	97	32.4	yes
C4	72	male	yes	no	ex	yes	no	no	2	180	93	28.7	yes
C5	72	male	yes	yes	no	yes	no	no	3	180	100	30.9	yes
C6	70	male	yes	no	no	yes	no	no	2	173	78	26.1	yes
C7	82	male	yes	yes	no	yes	no	no	3	179	86	26.8	yes
C8	73	male	yes	no	no	no	no	no	2	172	78	26.4	yes
C9	80	male	yes	no	ex	yes	no	no	3	182	100	30.2	yes
C10	79	male	yes	no	ex	yes	yes	no	3	182	90	27.2	yes
C11	77	female	yes	yes	no	yes	yes	no	3	169	62	21.7	no
C12	76	female	no	no	yes	yes	no	no	2	163	45	16.9	yes
C13	69	female	yes	no	no	yes	no	no	2	168	79	28.0	yes
C14	81	female	yes	no	no	yes	no	no	3	160	67	26.2	yes
C15	71	female	yes	yes	ex	yes	no	no	2	171	85	29.1	yes
C16	72	female	yes	no	ex	yes	no	no	2	150	54	24.0	yes
C17	62	female	yes	yes	no	yes	yes	no	2	156	98	40.3	yes
C18	88	female	yes	yes	no	yes	yes	no	3	165	58	21.3	yes
C19	81	female	yes	no	no	yes	yes	no	3	155	55	22.9	yes
C20	71	female	yes	no	no	no	no	no	2	162	78	29.7	yes
F1	56	male	yes	no	yes	yes	no	no	2	170	62	21.5	yes
F2	60	male	yes	no	yes	no	no	no	2	196	105	27.3	yes
F3	59	male	yes	yes	ex	yes	yes	no	3	172	75	25.4	yes
F4	59	male	yes	no	yes	no	yes	no	3	176	70	22.6	yes
F5	78	male	yes	no	yes	yes	no	no	3	170	69	23.9	yes
F6	69	male	yes	yes	ex	yes	no	no	3	175	85	27.8	yes
F7	64	female	yes	no	ex	no	no	no	2	163	62	23.3	yes
F8	75	female	yes	no	no	yes	yes	no	3	160	76	29.7	yes
F9	72	female	no	no	ex	yes	yes	no	3	156	47	19.3	no
F10	63	female	yes	no	ex	yes	no	no	2	175	56	18.3	yes
